# Chronic Episodic Hypotension as a Cause of Chronic Kidney Disease

**DOI:** 10.1155/carm/6651563

**Published:** 2025-07-01

**Authors:** Hong Phuc Nguyen, Brian Schlesinger, Julie Cordero, Anna Madorskaya

**Affiliations:** Department of Primary Care, Heritage Sierra Medical Group, Lancaster, California, USA

## Abstract

Chronic kidney disease (CKD) is a global health concern characterized by the gradual loss of renal function as seen by the decline of the estimated glomerular filtration rate (eGFR). While hypertension is a well-documented risk factor for CKD progression, the influence of hypotension on this condition remains less explored. Hypotension is an independent predictor of CKD progression. Patients experiencing hypotension show a significantly faster and steadier decline in the eGFR when compared to the normotensive CKD patients. Notably, the use of antihypertensive medication and diuretics is associated with a higher likelihood of hypotension. We have seen several cases where tapering down or completely stopping antihypertensive medications, as well as treating orthostatic hypotension, has resulted in improved CKD and renal function. These findings highlight the importance of monitoring blood pressure levels in CKD patients, as hypotension may contribute to an accelerated decline in renal function and increased morbidity. These case studies aim to investigate the sustained improvement of the eGFR from avoiding chronic episodic hypotension by adjusting blood pressure medications for a duration of 2–5 years.

## 1. Introduction

Chronic kidney disease (CKD) is defined as kidney damage where an estimated glomerular filtration rate (eGFR) is less than 60 mL/min/1.73 m^2^, persisting for 3 months or more [1]. Most clinicians have clinical protocols to treat acute kidney injury (AKI) secondary to hypotension from acute processes such as vomiting or diarrhea [2]. Hospital emergency departments and intensive care units worldwide see acute hypotension leading to AKI on a regular basis [3, 4]. However, clinicians are not recognizing CKD as a result of chronic episodes of hypotension, even though the only difference between the two concepts is chronicity. Chronic episodic hypotension tends to show up when a patient is on too much medication to control HTN, which may in turn worsen the status of kidney function. Episodic hypotension in older adults is common, as shown by a study of 588 elderly patients [5]. Hypertension treatment causing episodic hypotension was not insignificant in the Systolic Blood Pressure Intervention Trial (SPRINT) [6]. However, the current expectation is that treating hypertension will worsen kidney function, as seen from the analysis by Ku et al. and the SPRINT trial [7, 8]. The following cases demonstrate significant improvement in CKD by avoiding hypotensive blood pressure readings and symptoms. These patients have a duration of at least 3 years or more with eGFRs consistently less than 60.0 mL/min/1.73 m^2^.

## 2. Clinical Summary

We encouraged each patient to keep a log of blood pressure readings at home by checking at different times of the day. We asked patients to report any symptoms of hypotension, such as lightheadedness or dizziness with position changes, weakness, or fainting. We titrated down patients' blood pressure medications if there were any symptoms noted or blood pressure readings documented that are indicative of hypotension. We closely monitored patients throughout the process in order to regulate blood pressure and adjust medications. We calculated all patients' eGFRs using the 2021 CKD-EPI creatinine equation as recommended by the National Kidney Foundation and the American Society of Nephrology Task Force [9, 10].

**Figure 1:**
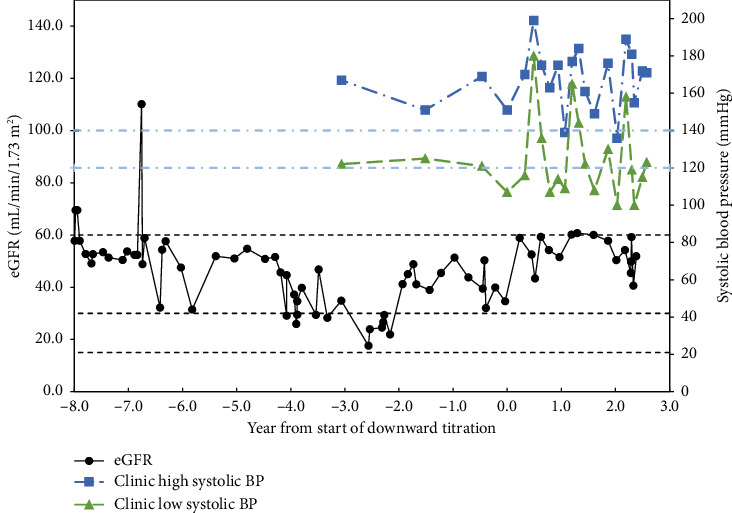
Patient 1.

Patient 1 is a 69-year-old Caucasian male with hypertension and a 6.5-year history of CKD. His eGFR average was 36.5 ± 10 mL/min/1.73 m^2^ with a nadir of 18 mL/min/1.73 m^2^ for the 3 years before implementing the protocol to avoid hypotension. After adjusting medication to avoid hypotension, his eGFR changed to 52.8 ± 6.4 mL/min/1.73 m^2^ and *p* < 0.001 for the past year (Figure 1). Hypertensive medications were adjusted from amlodipine 5 mg daily, losartan 50 mg daily, and carvedilol 25 mg twice daily to metoprolol succinate 50 mg twice daily and as needed and lisinopril 2.5 mg daily for systolic blood pressure above 160 mmHg.

**Figure 2:**
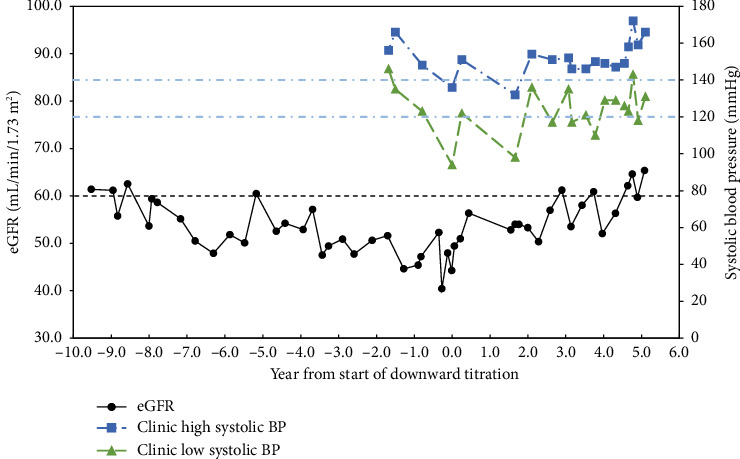
Patient 2.

Patient 2 is an 82-year-old Caucasian female with hypertension and a 4.5-year history of CKD. Her eGFR average was 47.7 ± 3.5 mL/min/1.73 m^2^ for the 3 years before implementing the protocol to avoid hypotension. After adjusting medication to avoid hypotension, her eGFR improved to 56.4 ± 4.8 mL/min/1.73 m^2^ and *p* < 0.001 for the past 5 years. For the past year, her eGFR has been averaging 60.1 ± 5.1 mL/min/1.73 m^2^ and *p* < 0.001 (Figure 2). Hypertensive medications were adjusted from valsartan 160 mg twice daily and propranolol 20 mg twice daily to midodrine 10 mg twice daily.

**Figure 3:**
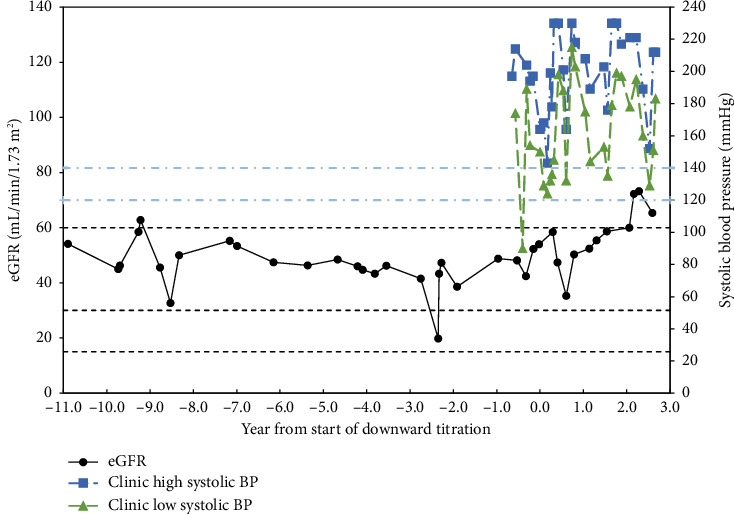
Patient 3.

Patient 3 is a 78-year-old Caucasian female with hypertension and an 8.5-year history of CKD. Her eGFR average was 45.2 ± 7.7 mL/min/1.73 m^2^ with a nadir of 20 mL/min/1.73 m^2^ for the 8.5 years before implementing the protocol to avoid hypotension. After starting to adjust medication 2.5 years ago, her eGFR changed to 65.9 ± 6.7 mL/min/1.73 m^2^ and *p* < 0.001 for the past year (Figure 3). For the first time in over 10 years, the patient started to have eGFR readings of 60 or more. Hypertensive medications were adjusted from amlodipine 10 mg daily, losartan 100 mg daily, and hydralazine 50 mg three times daily to amlodipine 1.25 mg daily and as needed and lisinopril 2.5 mg every evening for systolic blood pressure above 150 mmHg.

## 3. Discussion

Kidney function declines with age, with an estimation annually ranging from 0.4 to 1.8 mL/min/1.73 m^2^ for normal blood pressure and up to 2.4 mL/min/1.73 m^2^ for patients with untreated Stage 2 hypertension [11–13]. Most clinical trials do not expect to see end-stage kidney disease in their patients who started with Stage 3 CKD, given the relatively slow decline of kidney function in the natural progression of CKD. However, an analysis of the Atherosclerosis Risk in Communities Study that focused on orthostatic hypotension and incident CKD showed that there was an increased risk of CKD among persons with orthostatic hypotension compared to those without it [14]. Those findings suggested that orthostatic hypotension increases the risk of CKD in middle-aged persons. They also surmise that impaired glomerular autoregulation leading to decreased kidney perfusion and intraglomerular pressure may have a hand in the development of CKD.

Hypotension leading to decreased renal perfusion and resulting in kidney injury is a common concept. Clinicians worldwide treat many cases of acute hypotension on a daily basis. However, clinicians are not acknowledging chronic episodic hypotension as a cause of chronic kidney injury, even though it is a similar concept to acute hypotension except for chronicity. Many guidelines, including the Seventh Report of the Joint National Committee on Prevention, Detection, Evaluation, and Treatment of High Blood Pressure and the Kidney Disease Improving Global Outcomes 2012 Clinical Practice Guideline for the Evaluation and Management of CKD, warn about hypotension in the management of hypertension [15–17]. In practice, it is not uncommon for hypertensive treatment to cause hypotension. For example, the standard treatment group in the SPRINT trial had many hypotensive episodes, but clinicians did not adjust blood pressure medication dosages since they do not think that these results are significant [6].

The SPRINT trial concluded that orthostatic hypotension from hypertension treatment is frequent but not a reason to down-titrate therapy. However, since the standard treatment group also has frequent signs and symptoms of hypotension, the comparison does not rule out hypotension due to hypertension treatment as a contributor to negative outcomes [18]. Both the ACCORD trial and the SPRINT trial targeted systolic blood pressure of less than 120 mmHg, but only the SPRINT trial demonstrated the clear benefit of intensive treatment [6, 19]. When comparing the criteria for both trials regarding blood pressure, it is apparent that the difference is in the SPRINT trial's exclusion criterion of blood pressure less than 110 mmHg following 1 min of standing [20, 21]. This exclusion criterion removes a significant portion of patients who are at risk for significant complications from hypotension and keeps patients who are mildly hypotensive. Another difference between the two studies is that the standard treatment of the SPRINT trial has more signs and symptoms of hypotension compared to the ACCORD trial; furthermore, the standard treatment arm should only have rare incidents of overtreatment. A Cochrane review shows little to no benefit in total and cardiovascular mortality for lower blood pressure targets [22]. Lastly, Beddhu et al.'s review of both the ACCORD and the SPRINT trials shows that intensive hypertension treatment leads to increased incidents of CKD [23].

Although kidney function gradually declines with age, studies have shown potential for improvement. Using a 3-month duration for CKD, Liu et al. looked at a cohort of 81,320 patients, showing a 14%–19% probability of regression in 5 years [24]. Borrelli et al. presented a prospective study of 1418 patients with CKD of at least 1 year duration receiving nephrology care leading to improvement in kidney function [25]. However, neither of the above studies was using a specific intervention.

Tomlinson et al. presented a study of 98 patients with CKD Stages 3 and 4, where 70%–80% have at least one ambulatory blood pressure reading in the hypotensive range [26]. Even if avoiding hypotension only improved a fraction of these patients' kidney disease, the number of patients benefiting would be significant.

## 4. Conclusions

The kidney functions of the presented patients show a significant improvement with the specific intervention of avoiding signs and symptoms of hypotension. The prevalence of CKD 3 and 4 is 6.9% [27]. Applying this practice of avoiding low blood pressure can help many patients, since there is a large population of patients with CKD, and a significant portion of those have episodic hypotension, as indicated by blood pressure readings or symptoms. These patients will not only have their CKD better controlled but also have a better quality of life by eliminating symptomatic hypotension.

## Data Availability

The data that support the findings of this study are available from the corresponding author upon reasonable request.
